# Another Death from Anæsthesia

**Published:** 1874-01

**Authors:** 


### Editorial.

### Another Death from Anæsthesia.

Just as our last JOURNAL, in which we made some remarks concerning Ether and Chloroform, was going to press, we received copies of the *Boston Evening Journal* for Dec., 10th, 11th, 12th and 16th, 1873, containing an account of the Coroners inquest in a case of death from ETHER, occurring during an operation for Pelvic abscess, in Lynn, Mass. From all that we can learn on reading the testimony the ether was properly and carefully administered, although it leaves us to conjecture regarding the amount taken. A pound was sent for and after the death of the patient 1.728 fluid ounces were handed to Prof. E. S. Wood for analysis; that this was all that remained, the testimony does not state.

Our Boston friends have been too sanguine regarding the safety of ether and the danger which *always* accompanied the administration of chloroform.

It is far from our intention to advocate the other extreme and declare chloroform safe and ether unsafe. On the contrary we heartily agree with the Boston physicians regarding the greater safety of ether, but must qualify this opinion by saying that we regard neither as entirely free from danger,—both as highly dangerous unless administered *with care by experienced hands.* Boston medical authorities have been in the habit of decrying the use of chloroform, and looking upon physicians who were in the daily habit of using it as being entirely inexcusable, and should death occur, accountable for the accident. We have regarded their opinion as biased by custom and partially, perhaps by the fact that Ether was claimed as a Boston invention. We hope that the time will come when they will look upon this matter in its true light and not as one physician did in this examination, declare that "any amount of ether could be administered to a patient." As a sample of what prejudice will cause a man to say, we copy the following extract from the testimony of Dr. Francis Minot:

"Francis Minot was then called. My occupation is a physician; am an Assistant Professor at Harvard College; also a physician at the Massachusetts General Hospital; have had great experience in the use of ether; have never known a sudden death while the patient was under ether; think it would be a difficult matter to kill a healthy patient; think a person might be killed by asphyxia, but by no other way; do not think death could be produced by over anæsthesia; have never heard of a case where ether might cause the death of a debilitated person; have known a person kept under ether for any length of time; I think that chloroform can kill a person; this is done by a sudden paralyzation of the heart, which is unexpected; in most cases the patient is sitting up and the operation is a slight one; think that ether is generally administered in a careful manner; think from what I have heard of the late Mrs. Homan's case that she did not die from ether; think it must have been from chloroform; do not think any death has occurred in the hospital from the administration of ether."

If the newspaper report of the testimony can be depended upon, it is not only highly laughable but absurd.

The testimony of other witnesses showed that the patient had repeatedly been under the influence of chloroform without any unpleasant results.

The following testimony of Dr. L. F. Warner, reads much more as if he had carefully considered the subject:

"L. F. Warner next testified. Live in Boston; am by occupation a physician; have made a specialty of diseases of women; have had experience with the use of ether; think, if administered in a proper manner, it can produce death; it produces death by over-anæsthesia; think that chloroform has no peculiar properties in itself to cause death; if chloroform in a case produces death, think it is due to the condition of the patient; a person, by an overdose of chloroform, can be killed as well as by an overdose of alcohol; think a person can be killed in the same way with ether; cannot say that ether is administered carelessly by physicians; the signs of asphyxia would be a lack of breathing caused by ether given so rapidly as to exclude all air from the patient; do not believe with Dr. Wheeler that any amount of ether could be administered to a patient; think that ether is safer than chloroform; could not say that ether is administered too carelessly in Boston or New England; think that the doctrine that ether cannot kill a dangerous one; have seen many cases of pelvic abscess; in Mrs. Homan's case would not have attempted an operation without the aid of ether; would have greatly urged the operation: was present at the autopsy on Mrs. Homan's body and from that examination could see no caus for her death; as an expert I should say that she died from the ether; do not think the amount given was too much; the operation on Mrs. Homan's was properly performed; that could not have positively conduced to her death; think that there would have not been immediate death from the operation if ether had not been administered; from the evidence I see no evidence of the doctors forcing ether upon the patient; think it very easy for a non-professional person to imagine they felt the beating of a pulse; think the twitching of muscles would deceive a person; do not believe that Mrs. Homan was alive after Drs. Bixby and Graves announced that she was dead."

The latter portion of this testimony refers to the statement of a Dr. (?) Cushing, that he was called about an hour after Drs. Bixby and Graves had left the case, and found the heart still pulsating and slight pulse at the wrist. Altogether the case is very interesting. We append the verdict of the Coroner's Jury, which reads as if they wished to shield the Ether from as much blame as possible. The Jury found:

"That the said Elizabeth M. Homer, came to her death at her residence, 7 Pleasant St., Lynn, on the fourth day of Dec., 1873 between the hours of four and five o'clock, P M., from the combined effect of sulphuric Ether and nervous exhaustion, while undergoing a trifling surgical operation; and the jury further find that the etherization and operation were properly done, and that prompt, energetic and all necessary means were employed to resuscitate the patient."

With the verdict in the case of death from Chloroform and Ether in his mind, no Doctor in Boston will dare to administer chloroform to a patient in the future, and should he be unfortunate enough to have a patient die under the influence of Ether, he can lay the death to the influence of nervous exhaustion.

